# Everybody's Page

**Published:** 1890-07-26

**Authors:** 


### Everybody's Page.

The total annual home consumption of opium in China has lately been reckoned to be about 41,800,000 pounds.

It is worthy of note that the inaugural meeting of the Universal *Peace* Congress opened with *war!* One hot discussion arose as to why certain delegates were excluded, and another as to whether the daily proceedings were to open with prayer.

Professor MacIvor, if we are to believe the testimony of some of the leading chemists, has done a great thing for health as well as for trade. His new white lead process produces in hours what it formerly took months to effect, and the cost is much less. As regards health, the process is a wet one, so that there is no dust, and so no danger from that source.

The report just issued shows that the number of entries on Sundays at the People's Palace Library was registered at 27,228. The library is only open on Sundays from three to ten p.m. How about the indifference of the working classes as regards Sunday opening of museums, libraries, &c.? Great demand is made for technical and scientific works, and the class of reading is improving much.

If only the negotiations now on foot with the Postmaster-General for the establishment of a system of electric calls could be brought to a successful issue, the Boy Messengers' Company would be a really useful affair; but at present the usefulness of the institution is much marred by the inability of the public to get a messenger without sending to the depôt for one.

That some good may come and a solid, definite control over baby-farming and children's life assurance be the result of the Select Committees now inquiring into the protection of children's lives, is our earnest hope. Five years' penal servitude was what Thomas Lloyd got at Sheffield the other day for starving and brutally thrashing a child. Physical pain such as they have given is what ought to be meted out to such brutes as these, and a man who thrashes should be thrashed.

The first number of the *Gentlewoman* has been kindly sent to us, and we cordially wish her a long life and a happy one. One of her promises appeals strongly to us—she says, "Though recording all news and events, she will never descend to trivial or ill-natured personalities." She will tell of the doings of philanthropists, artists, poets, sportswomen—in fact, all sorts and conditions of women; and here we commend the *Gentlewoman* again, for dress, fashion, and society are, thank goodness, not the sole subjects of interest to women nowadays.

The new train de luxe which is to run from Paris to Brindisi in connection with the P. and O. steamship mail services to India and China is one more step in luxurious travelling. Though globe-trotting is of every-day occurrence, and a visit to India part of a rich man's programme, increased experience of the sea does not, alas, bring decreased experience of sea-sickness, and the overland route is a godsend to many. To these it will be joy to know of a train in which they can leave London on Fridays at a quarter-past three. At Calais they get into a train which possesses luxurious sleeping accommodation for fifty persons, and a restaurant car of the most perfect description, and without changing carriages all the way and without any extra charge over the present rates, the lucky passenger will arrive at Brindisi at four p.m. on Sunday. This will be a great improvement on the old service, which carried mails as well as passengers.

Lifts are still by no means common luxuries in England, but if the new motive power, compressed air, continues successful, we shall find them in far more general use; for the expense of the new method is said to be one-half that of hydraulic lifts.

It is very difficult to knock down an idea when it has once taken firmly hold of the public. Koch and others have demonstrated that burning sulphur is useless for disinfection. Sulphur may kill bacteria but it does not kill spores. Disease germs adhere most closely to woollen materials and sulphur does not penetrate woollen. But steam as it comes out of a kettle at 212 F. will in fifteen minutes destroy any spores.

A writer in the *New York Medical Record* protests against the prevalent wholesale prescription of spectacles, particularly for young people and children, with insufficient cause. The same writer insists, too, on what we have so often suggested in these pages, that so much might be done if children were made to sit with their work at a proper distance from them and the light were properly arranged.

The Earl of Meath is evidently one of those who knows how fruitless it is to develop the mind and leave the body undeveloped. Therefore, he proposes a Bill for Physical Education in Elementary schools, and we cordially wish him success in improving the physique of our London children. But a little more food and a little less "crowding" is the legislation most of the children want in order to develop.

The punkah-like use of the fan, tapping the floor in time to music, or softly blowing through the teeth, are some of the irritating tricks due to "nerves," so a writer in the *Globe* thinks. What we should like to impress upon the minds of "nervous" people is, that what they suffer is absolutely nothing to the pangs and sufferings they cause others. Nerves like weeds grow a-pace if not kept under, and if every selfish, fidgetting trick is to be sympathized with as caused by them, "nervous" folk are likely to be on the increase.

Dr. Spratling, of New Jersey, is of opinion that the word "asylum," as descriptive of a place of reception and treatment of the insane, ought to be replaced by "hospital." The term "hospital" serves better to show the character of the institution, and impress upon the public the fact so continuously ignored of the physical basis of insanity and all that that simple doctrine implies. To be "shut up in an asylum" is a horror to a sensitive mind, to be "treated in a hospital" a natural thing. Dr. Spratling thinks that as insanity is now generally conceded to be a physical disease, and as such a malady for treatment, that the word "hospital" ought to be in general use for these institutions.

It is argued by some people that Addison did no good in his time by his contributions to the *Spectator*, that in short the writings of a man who only laughed at the fashions and follies of the world, and who never treated them seriously, would be enjoyed for the moment and never reflected upon afterwards. It strikes us, however, that if another Addison were to appear now, touching with the same lightness on the freaks and fancies of this century, and yet with the undercurrent of reproach which to us seems to run all through the *Spectator*, the good he would achieve would be anything but inconsiderate. Addison laughed at the foolish belles and beaux "who complain of the shortness of time and yet have much more than they know what to do with." He knew they would listen to nothing very serious. And he knew, too, that the saints need no reforming.

